# Application of diffusion tensor imaging analysis along perivascular spaces in evaluating glymphatic system function in type 2 diabetes mellitus

**DOI:** 10.3389/fnins.2026.1801155

**Published:** 2026-03-23

**Authors:** Yujuan Wang, Yang Ji, Guangxu Han, Ya Li, Jin Yan, Yuanyuan Huo, Xiaoqing Liu, Shuanghong Zhang

**Affiliations:** 1 Department of Radiology, The First Affiliated Hospital of Xi'an Medical University, Xi'an, Shaanxi, China; 2 School of General Medicine, Xi'an Medical University, Xi'an, Shaanxi, China; 3 GE HealthCare MR Research, Beijing, China; 4 Department of Endocrinology, The First Affiliated Hospital of Xi'an Medical University, Xi'an, Shaanxi, China

**Keywords:** brain glymphatic system, diffusion tensor imaging analysis along perivascular spaces, insulin resistance, mediation analysis, montreal cognitive assessment, type 2 diabetes mellitus, white matter Fazekas score

## Abstract

**Background:**

Type 2 diabetes mellitus (T2DM) is often accompanied by cognitive dysfunction, though its underlying mechanisms remain unclear. Impaired glymphatic system function may contribute to cognitive impairment. Herein, we aimed to evaluate glymphatic activity in patients with T2DM using diffusion tensor imaging analysis along perivascular spaces (DTI-ALPS) and to explore its mediating role between white matter hyperintensities (Fazekas score) and Montreal Cognitive Assessment (MoCA) scores.

**Methods:**

Thirty-eight patients with T2DM and 54 age- and sex-matched healthy controls underwent 3.0 T magnetic resonance imaging (MRI) and MoCA testing. The DTI-ALPS index was derived from diffusion tensor imaging data. Clinical variables included demographics, disease duration, metabolic indicators, and the homeostatic model assessment of insulin resistance (HOMA-IR) index. Group differences were analyzed using ANCOVA, correlations were assessed using Pearson analysis, and mediation analysis was used to evaluate the role of the DTI-ALPS index between Fazekas score and MoCA.

**Results:**

The DTI-ALPS index was significantly lower in patients with T2DM having cognitive impairment compared to both healthy controls and patients with T2DM without cognitive impairment (*p* < 0.001). MoCA scores positively correlated with the DTI-ALPS index (*r* = 0.860, *p* < 0.001), while HOMA-IR showed a negative correlation (*r* = −0.326, *p* < 0.05). Longer disease duration and higher Fazekas scores were associated with reduced DTI-ALPS index (*p* < 0.05). Mediation analysis indicated that the association between Fazekas score and MoCA was significantly and partially accounted for by the DTI-ALPS index in patients with T2DM (effect = −0.344, *p* < 0.05).

**Conclusion:**

The DTI-ALPS index was strongly associated with cognitive performance in patients with T2DM. Mediation analysis suggests that this relationship may reflect a mechanism in which impaired glymphatic function mediates the impact of white matter lesions on cognitive decline.

## 1 Introduction

Type 2 diabetes mellitus (T2DM) is a chronic metabolic disorder characterized by hyperglycemia, with typical features including insulin resistance and/or insufficient insulin secretion. It is recognized as an independent risk factor for cognitive impairment (Cukierman-Yaffe et al., 2021), significantly affecting quality of life. However, it is crucial to acknowledge that cognitive impairment in T2DM is multifactorial and not exclusive to the diabetic state. Age remains the strongest overall risk factor, followed by other contributors such as educational attainment (Livingston et al., 2020), vascular comorbidities, and cerebrovascular damage, including white matter disease (Wardlaw et al., 2013). Compared to patients with T2DM without cognitive impairment (T2DM-nCI) and healthy controls (HC), patients with T2DM and cognitive impairment (T2DM-CI) often exhibit more pronounced abnormalities in brain structure and function. Although mechanisms such as insulin resistance, neuroinflammation, and microvascular dysfunction have been implicated (Luo et al., 2022), the pathophysiology underlying diabetes-related cognitive decline remains incompletely understood and is likely intertwined with these other age- and vascular-related pathways. This study does not seek to establish causality or claim exclusivity for diabetes; rather, it aims to investigate one specific mechanistic pathway within this complex etiological landscape.

The glymphatic system serves as a key brain-wide waste clearance mechanism for proteins like beta-amyloid and tau, thereby linking the maintenance of neural homeostasis directly to the pathophysiology of neurodegenerative diseases (Beschorner and Nedergaard, 2024). Studies show that glymphatic function in Alzheimer’s disease (AD) is significantly impaired, preceding both cognitive decline and the appearance of typical pathological changes (Hsu et al., 2023). In mild cognitive impairment (MCI), glymphatic dysfunction is considered a risk factor for the progression of cognitive impairment (Bao et al., 2025), while in Parkinson’s disease (PD), it correlates with disease severity and motor complications (He et al., 2023). These findings suggest that glymphatic system assessment may serve as a biomarker for early diagnosis and disease monitoring in neurodegenerative conditions.

Previous studies on the brain glymphatic system mainly relied on animal experiments (Iliff et al., 2012); human glymphatic system assessment required intrathecal injection of contrast agents (Lee et al., 2021) and observation of the exchange process between cerebrospinal fluid (CSF) and interstitial fluid (ISF) using dynamic contrast-enhanced magnetic resonance imaging (DCE-MRI), all of which are invasive methods. In 2017, Taoka et al. (2017) proposed a diffusion tensor imaging analysis method based on perivascular spaces (DTI-ALPS) as a non-invasive technique to evaluate activity of the glymphatic system, with good patient compliance. Zhang et al. (2021) demonstrated a significant correlation between the DTI-ALPS index and the classical DCE-MRI method, supporting its feasibility for assessing glymphatic system function.

The DTI-ALPS index obtained through this method can directly quantify the function of the glymphatic system and has emerged as a promising neuroimaging biomarker. The DTI-ALPS index has been applied to various diseases, such as AD and PD (Taoka et al., 2024). Previous studies related to T2DM with cognitive impairment (T2DM-CI) have confirmed that white matter hyperintensities (WMHs) are strongly associated with disease progression and are recognized as biomarkers of brain injury with significant clinical and research value (Wang et al., 2020). However, their role in glymphatic dysfunction in T2DM-CI remains unclear.

In this study, we investigate the functional changes of the glymphatic system in T2DM-CI, primarily conducting research in three aspects:

(i) To explore the changes in DTI-ALPS index among patients with T2DM with different disease durations. Longer disease duration may exacerbate glymphatic dysfunction due to cumulative effects of hyperglycemia and insulin resistance. Clarifying the association between disease duration and DTI-ALPS index can provide a basis for risk stratification of T2DM-CI.
(ii) To analyze the correlation between the insulin resistance index (HOMA-IR) and DTI-ALPS. Insulin resistance is not only a core pathological feature of T2DM but can also interfere with the clearance process of metabolic waste in the glymphatic system through pathways such as affecting blood–brain barrier permeability and inhibiting astrocyte function (Padovani et al., 2025). Elucidating their association helps reveal the role mechanism of insulin resistance in glymphatic system damage in T2DM-CI.
(iii) To investigate the correlation between WMH and DTI-ALPS, and to explore the mediating effect of the DTI-ALPS index between WMH (Fazekas scores) and MoCA cognitive scores in patients with T2DM. WMHs are common manifestations of cerebral small vessel disease in T2DM (Wang et al., 2020), essentially representing microstructural damage of white matter and disruption of the blood–brain barrier (Serlin et al., 2011). The function of the glymphatic system depends on intact white matter structure and blood–brain barrier function (Jessen et al., 2015). Correlation analysis between the two can clarify the mutual interaction between white matter injury and glymphatic system dysfunction.

We propose the following hypotheses: (i) DTI-ALPS index is lower in patients with T2DM compared to HC, with the greatest reduction in those with cognitive impairment. (ii) DTI-ALPS index is associated with white matter damage, insulin resistance, and cognitive dysfunction, mediating the relationship between Fazekas scores and cognitive decline in T2DM.

## 2 Materials and methods

### 2.1 General information

This study is a cross-sectional case–control study, and the sample size was determined based on previous literature research. This study initially enrolled 38 patients with type 2 diabetes mellitus (T2DM) and 54 age- and sex-matched healthy controls (HC) underwent 3.0 T magnetic resonance imaging (MRI) and the Montreal Cognitive Assessment (MoCA). Subsequently, the T2DM group was further subdivided into T2DM-CI and T2DM-nCI based on MoCA scores. The DTI-ALPS index was derived from diffusion tensor imaging (DTI) data. Clinical variables included demographics, disease duration, metabolic indicators, and the homeostatic model assessment of insulin resistance (HOMA-IR).

Inclusion criteria: Diagnosis consistent with the ‘Chinese Guidelines for the Prevention and Treatment of Diabetes Mellitus (2025 Edition) (Jia et al., 2025); no contraindications to MRI; ability to complete MRI smoothly with good image quality; intact consciousness, Glasgow Coma Scale (GCS) was formally assessed; no history of cranial trauma, cerebral hemorrhage, cerebral infarction, brain tumor, or radiotherapy/chemotherapy.

Exclusion criteria: Patients with type 1 diabetes or other specific types of diabetes; contraindications to MRI; impaired consciousness; history of cranial trauma, cerebral hemorrhage, cerebral infarction, brain tumor, radiotherapy, or chemotherapy; systemic diseases.

**Figure 1:**
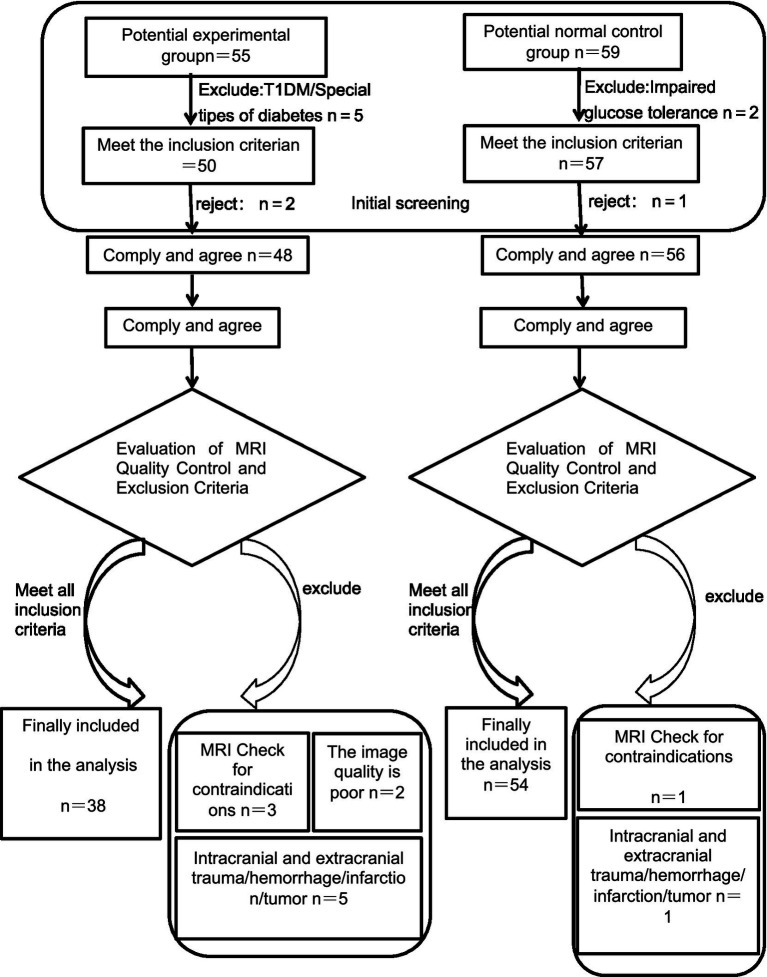
Flowchart showing the selection criteria for the study.

The study flow chart is shown in Figure 1. All procedures complied with the ethical standards of the institutional ethics committee and were approved accordingly.

### 2.2 Research methods

#### 2.2.1 Clinical data collection

All participants underwent MoCA cognitive assessment. Patient general information was recorded, including age, sex, body mass index (BMI), years of education, and disease duration. Laboratory indicators included fasting blood glucose, fasting insulin, glycated hemoglobin, and C-peptide. Insulin resistance was evaluated using the HOMA-IR index, calculated according to the formula: HOMA−IR=fastingbloodglucose\mathrm{×}fastinginsulin/22.5 (Matthews et al., 1985), where the unit of fasting blood glucose is mmol/L and the unit of fasting insulin is mIU/L. Patients were categorized as follows:

- Disease duration: <10 years (19 cases) and ≥10 years (19 cases).
- Insulin resistance: HOMA-IR ≥ 2.69 (20 cases) vs. HOMA-IR < 2.69 (18 cases).
- Cognition (MoCA-BJ): Maximum score = 30 (Yu et al., 2012).
  - Grade 0 (normal cognition): ≥26 points (10 cases).
  - Grade 1 (mild impairment): 18 ≤ *x* < 25 (11 cases).
  - Grade 2 (dementia): *x* ≤ 17 (17 cases).

For analysis, Grade 0 was classified as the normal cognition group, while Grades 1–2 were grouped as cognitive impairment. Demographic, neuropsychological, and MRI parameters of the T2DM and HC groups are presented in Table 1.

**Table 1:** Demographic, neuropsychological, and MRI parameters of the T2DM and HC groups.

Variable	HC	T2DM	PFDR	T2DM-nCI	T2DM-CI	PFDR
Age (years)	48.56 ± 13.65	62.37 ± 11.61	<0.001^a^	61.90 ± 5.59	62.54 ± 13.20	0.884^a^
Male	21	20	0.192^b^	5	15	0.846^b^
Education	12.11 ± 4.01	9.42 ± 3.75	0.002^a^	10.80 ± 2.90	8.93 ± 3.93	0.179^a^
BMI	23.77 ± 2.97	23.19 ± 2.65	0.342^a^	24.29 ± 1.81	22.80 ± 2.82	0.129^a^
MoCA score	26.07 ± 3.17	18.03 ± 8.24	<0.001^a^	27.50 ± 1.08	14.64 ± 6.90	<0.001^a^
Fazekas score	0.78 ± 0.66	1.39 ± 0.82	<0.001^a^	1.50 ± 0.71	1.36 ± 0.87	0.644^a^

a *p*-value was obtained using the two-sample *t*-test.

b *p*-value was obtained using the *χ*^2^ test.

Values are reported as the mean ± standard deviation for quantitative variables and as the frequency (percentage) for categorical variables. MRI, magnetic resonance imaging; T2DM, type 2 diabetes mellitus; HC, healthy controls; FDR, false discovery rate; T2DM-nCI, T2DM without cognitive impairment; T2DM-CI, T2DM with cognitive impairment; BMI, body mass index; MoCA, Montreal cognitive assessment.

#### 2.2.2 MRI data acquisition

All participants underwent scanning with the same 3.0-T MRI scanner (SIGNA Architect; GE HealthCare, Chicago, IL, USA) equipped with a 32-channel coil. Foam pads were used to fix the head, and participants were instructed to remain quiet to minimize movement, while wearing noise-reducing headphones. A 3.0 T superconducting MRI was employed to acquire the cranial brain DTI sequences and routine MRI scans. The DTI scanning parameters were as follows: TR/TE = 10,846 ms/85.5 ms, 15 diffusion gradient directions, *b*-value of 1,000 s/mm^2^, and voxel size of 2.0 mm × 2.0 mm × 2.0 mm. The parameters for routine scanning were as follows: FLAIR sequence (TR/TE = 8,000 ms/90 ms), field of view (FOV) of 23 mm × 23 mm, slice thickness/slice gap of 5.5 mm/1.0 mm, and 22 slices.

#### 2.2.3 DTI-ALPS index calculation

The DTI-ALPS index is calculated using a semi-automated and highly reliable data processing workflow based on diffusion-weighted (DW) images developed by Taoka et al. (2017).

The processing pipeline was as follows:

- DTI data processed with FSL (v6.0.3+) and MRtrix3.
- Denoising with MRtrix3’s *dwidenoise*; Gibbs ringing correction with *mrdegibbs*.
- Brain masks extracted from b0 images using FSL BET.
- Eddy-current and motion correction with FSL EDDY.
- Diffusion tensors fitted with FSL *dtifit* to derive fractional anisotropy (FA), principal eigenvector (V1), and tensor components (dxx, dyy, dzz).
- FA and tensor maps registered to the JHU-ICBM FA template using FLIRT and FNIRT.

At the axial plane of the body of the lateral ventricle, the evaluators manually placed four rectangular regions of interest (ROIs, measuring 5 mm × 5 mm, corresponding to approximately 10 voxels per direction) on the color-coded fractional anisotropy (FA) map, while remaining blinded to all participant-related information. Four regions of interest were defined: the left and right superior corona radiata (projection fibers) and the left and right superior longitudinal fasciculus (association fibers). These ROIs were used to compute the DTI-ALPS index, which reflects diffusivity along perivascular spaces relative to orthogonal directions. Specifically, diffusivity along the *x*-axis was averaged across projection and association fiber ROIs, while diffusivity along the *y*- and *z*-axes was averaged across the same ROIs, and the DTI-ALPS index was calculated as: $\backslash \text{text}\{\mathrm{DTI}-\text{ALPS index}=\text{mean}\;(\begin{array}{l} \text{Dxproj},\\ \text{Dxassoc} \end{array})/\text{mean}\;(\begin{array}{l} \text{Dyproj},\\ \text{Dzassoc} \end{array}).\}$ . The intraclass correlation coefficient (ICC) for the DTI-ALPS index demonstrated excellent reliability, with all values exceeding 0.90.

### 2.3 Assessment of WMHs

WMHs, markers of cerebrovascular disease, appear as low or isointense signals on T1-weighted imaging and hyperintense signals on T2-weighted FLAIR imaging. Severity was graded using the modified Fazekas scale (Fazekas et al., 1987):

- Grade 0: normal
- Grade 1: punctate hyperintensities
- Grade 2: patchy hyperintensities with partial fusion
- Grade 3: confluent hyperintensities indicating severe lesions

In this study, Grades 0–1 were grouped together (23 cases), and Grades 2–3 were grouped together (15 cases). MRI images were independently assessed by two blinded radiologists.

### 2.4 Statistical analysis

All statistical analyses were conducted in SPSS 27.0, with data summarized as mean ± standard deviation. Intergroup comparisons were performed with independent-sample *t*-tests for continuous data and chi-square tests for categorical data. Differences in the DTI-ALPS index across the normal control, T2DM without cognitive impairment, and T2DM with cognitive impairment groups were assessed by ANCOVA, ANCOVA included both age and education level as covariates. Independent-sample *t*-tests were further applied to compare the DTI-ALPS index across subgroups defined by HOMA-IR, diabetes duration, and white matter Fazekas scores in patients with T2DM. A mediation analysis evaluated whether the DTI-ALPS index mediated the relationship between WMHs (Fazekas score) and MoCA scores by Process Macro v3.5. Correlation analyses (Pearson for continuous) were conducted to assess the relationships between the DTI-ALPS index and HOMA-IR/MoCA scores, as well as between MoCA scores and diabetes duration/Fazekas scores. Finally, logistic regression analyzed the association between HOMA-IR and MoCA scores. All tests were two-tailed, with a *p*-value < 0.05 deemed statistically significant. Given the exploratory nature of the study, no correction for multiple comparisons was performed; therefore, the results should be interpreted with caution.

## 3 Results

### 3.1 Association between MoCA scores and related indicators

**Figure 2:**
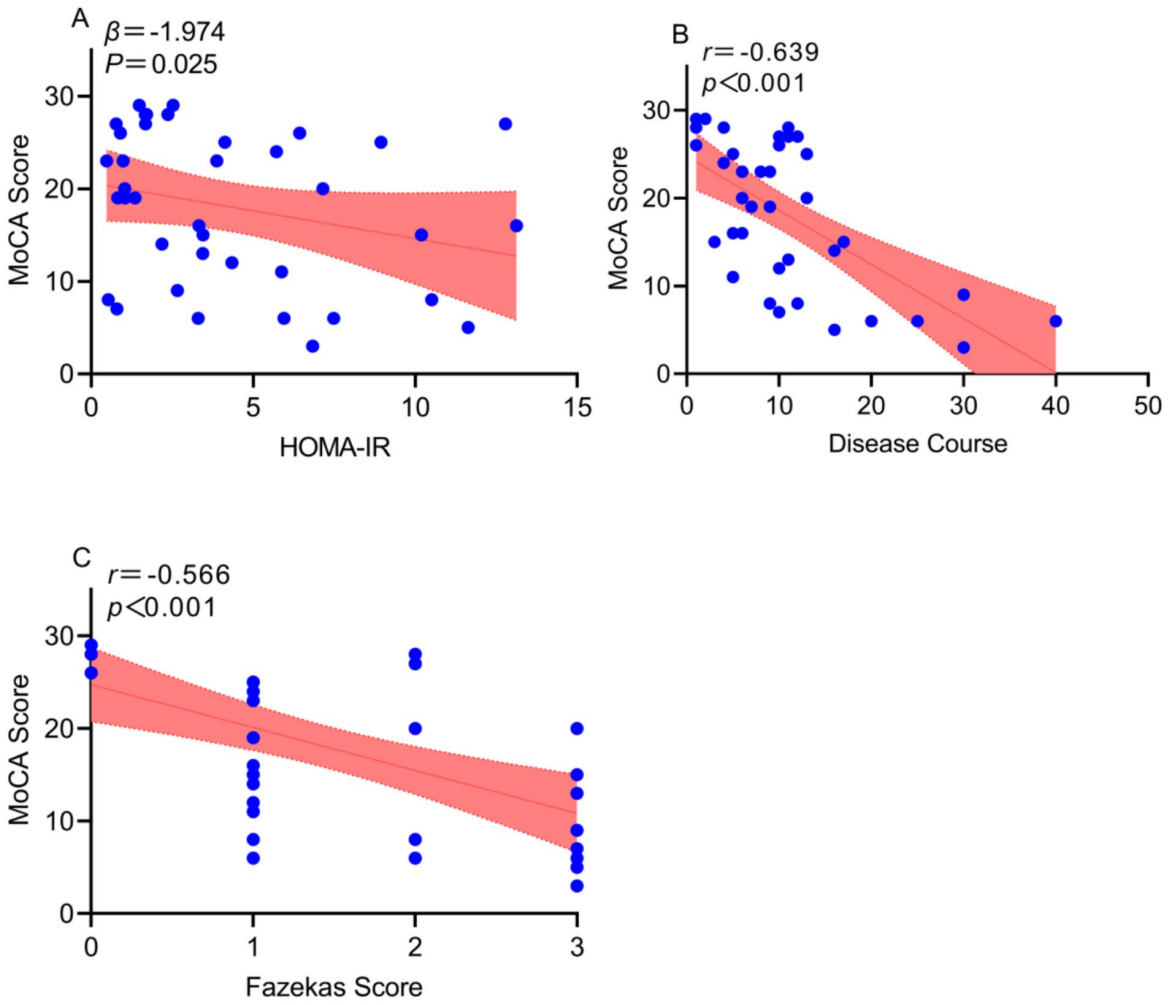
Scatter plot showing correlation between MoCA cognitive scores and related indicators. **(A)** Logistic regression analysis revealed a negative correlation between MoCA cognitive scores and HOMA-IR. **(B,C)** Pearson correlation analysis demonstrated that MoCA scores were significantly and negatively correlated with disease duration and Fazekas scores. The shaded areas denote the 95% confidence intervals, and the central curves represent the fitted linear correlation lines. Abbreviations: MoCA, Montreal Cognitive Assessment; HOMA-IR, Homeostasis Model Assessment of Insulin Resistance.

In the T2DM group, MoCA scores were correlated with HOMA-IR index, disease duration, and Fazekas score (*p* = 0.025, *p* < 0.001, *p* < 0.001, respectively) (Figure 2).

### 3.2 Comparison of the DTI-ALPS index across the HC, T2DM-nCI, and T2DM-CI groups

**Figure 3:**
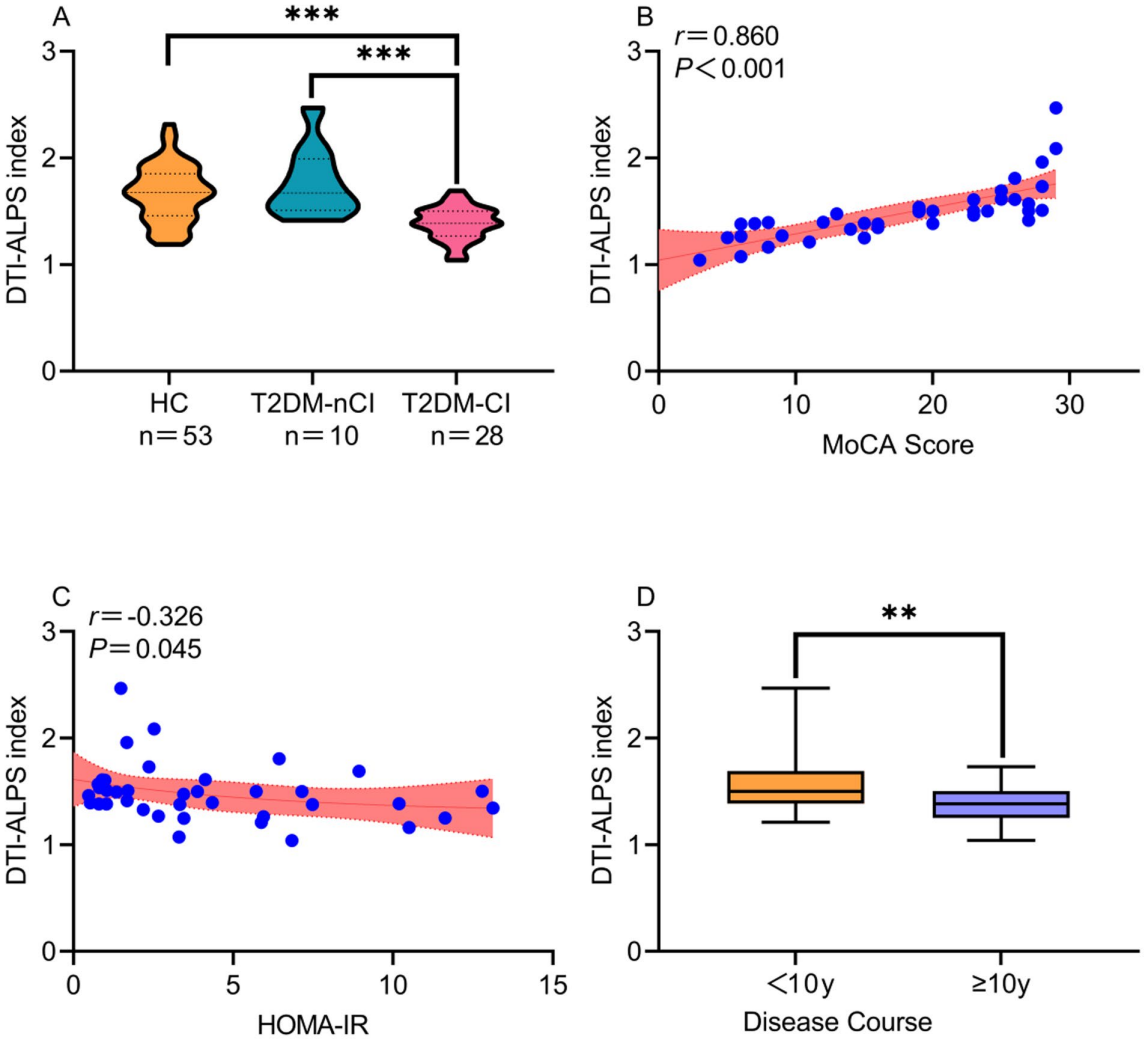
Correlation between DTI-ALPS index and related indicators. **(A)** Violin plot illustrating differences in the DTI-ALPS index among the HC group, T2DM-nCI group, and T2DM-CI group, compared using ANCOVA analysis of variance. **(B,C)** Scatter plot: Pearson correlation analysis demonstrated that MoCA scores positively correlated with DTI-ALPS index (*r* = 0.860, *p* < 0.001), while HOMA-IR showed a negative correlation (*r* = −0.326, *p* = 0.045). Shaded area represents the 95% confidence interval, and the middle curve represents the linear correlation coefficient. **(D)**. Box plot: Correlation between DTI-ALPS index and disease course was analyzed using independent samples *t*-test. For the box plot: the central line denotes the median, while the upper and lower box boundaries correspond to the upper and lower quartiles, respectively. T2DM, type 2 diabetes mellitus; HC, healthy controls; T2DM-nCI, T2DM without cognitive impairment; T2DM-CI, T2DM with cognitive impairment; MoCA, Montreal Cognitive Assessment; HOMA-IR, Homeostasis Model Assessment of Insulin Resistance; DTI-ALPS, diffusion tensor imaging–along the perivascular space.

Comparative analysis revealed that the DTI-ALPS index was significantly reduced in the T2DM-CI group relative to both the control group and the T2DM-nCI group (*p* < 0.001), as illustrated in Figure 3A (violin plot). Furthermore, MoCA scores demonstrated strong positive correlations with the DTI-ALPS index across all measurements (average *r* = 0.860, *p* < 0.001; left hemisphere *r* = 0.787, *p* < 0.001; right hemisphere *r* = 0.673, *p* < 0.001; see Table 2 and Figure 3B). These findings robustly support the association between the DTI-ALPS index and cognitive impairment in patients with T2DM.

**Table 2:** Correlation between MoCA cognitive score and DTI-ALPS index.

Statistic	Average DTI-ALPS	Left DTI-ALPS	Right DTI-ALPS
*r*-value	0.860	0.787	0.673
*p*-value	<0.001	<0.001	<0.001

Pearson correlation analysis; MoCA, Montreal cognitive assessment; DTI-ALPS, diffusion tensor imaging–along the perivascular space.

### 3.3 Relationship of insulin resistance with the DTI-ALPS index

The HOMA-IR index was negatively correlated with the DTI-ALPS index (*r* = −0.326, *p* = 0.045; Figure 3C).

Comparative analysis revealed significantly lower DTI-ALPS index in the HOMA-IR group across all measures: mean (1.386 ± 0.196 vs. 1.595 ± 0.301, *p* = 0.015), left-sided (1.401 ± 0.241 vs. 1.600 ± 0.284, *p* = 0.025), and right-sided values (1.371 ± 0.233 vs. 1.590 ± 0.448). While the right-sided difference did not reach statistical significance, both mean and left-sided DTI-ALPS index showed significant reductions in the HOMA-IR group (Table 3).

**Table 3:** Relationship between insulin resistance and DTI-ALPS index.

Region	HOMA-IR	Non-HOMA-IR	*t*-value	*p*-value
Average DTI-ALPS	1.386 ± 0.196	1.595 ± 0.301	−2.557	0.015
Left DTI-ALPS	1.401 ± 0.241	1.600 ± 0.284	−2.331	0.025
Right DTI-ALPS	1.371 ± 0.233	1.590 ± 0.448	−1.919	0.063

Values are reported as the mean ± standard deviation for quantitative variables. The *p*-value was obtained using the two-sample *t*-test. HOMA-IR, homeostasis model assessment of insulin resistance; DTI-ALPS, diffusion tensor imaging–along the perivascular space.

### 3.4 Comparative analysis of DTI-ALPS index across different stages of T2DM

Patients with shorter disease duration demonstrated consistently higher DTI-ALPS index across all measures: mean (1.591 ± 0.302 vs. 1.380 ± 0.186, *p* = 0.014), left-sided (1.623 ± 0.299 vs. 1.367 ± 0.184, *p* = 0.003), and right-sided values (1.558 ± 0.441 vs. 1.392 ± 0.251) (Table 4).

**Table 4:** Comparison of DTI-ALPS index among different disease durations of T2DM.

Region	<10 years	≥10 years	*t*-value	*p*-value
Average DTI-ALPS	1.591 ± 0.302	1.380 ± 0.186	2.595	0.014
Left DTI-ALPS	1.623 ± 0.299	1.367 ± 0.184	3.157	0.003
Right DTI-ALPS	1.558 ± 0.441	1.392 ± 0.251	1.427	0.162

Values are reported as the mean ± standard deviation for quantitative variables. The *p*-value was obtained using the two-sample *t*-test. T2DM, type 2 diabetes mellitus; DTI-ALPS, diffusion tensor imaging–along the perivascular space.

Boxplot analysis of DTI-ALPS index stratified by T2DM disease duration (<10 vs. ≥10 years) revealed consistent reductions in glymphatic function with longer disease duration (Figure 3D). The <10-year group demonstrated significantly higher distribution metrics across all measures—including upper quartile, median, and extreme values—compared to the ≥10-year group. Furthermore, disease duration showed a significant negative correlation with MoCA scores (*r* = −0.639, *p* < 0.001; Figure 2B), suggesting that progressive glymphatic dysfunction (as reflected by declining DTI-ALPS index) may contribute to worsening cognitive performance in chronic T2DM.

### 3.5 Association between Fazekas score and DTI-ALPS index

Glymphatic function, as measured by the DTI-ALPS index, was consistently superior in the Fazekas 0–1 group across all measures: mean (1.559 ± 0.309 vs. 1.372 ± 0.138, *p* = 0.035), left-sided (1.558 ± 0.320 vs. 1.399 ± 0.163), and right-sided values (1.560 ± 0.426 vs. 1.345 ± 0.184) (Table 5).

**Table 5:** Relationship between Fazekas score and DTI-ALPS index in T2DM.

Region	0–1 point	2–3 points	*t*-value	*p*-value
Average DTI-ALPS	1.559 ± 0.309	1.372 ± 0.138	2.193	0.035
Left DTI-ALPS	1.558 ± 0.320	1.399 ± 0.163	1.767	0.086
Right DTI-ALPS	1.560 ± 0.426	1.345 ± 0.184	1.839	0.074

Values are reported as the mean ± standard deviation for quantitative variables. The *p*-value was obtained using the two-sample *t*-test. T2DM, type 2 diabetes mellitus; DTI-ALPS, diffusion tensor imaging–along the perivascular space.

### 3.6 Mediation analysis of DTI-ALPS index in the relationship between Fazekas score and MoCA score in T2DM

**Figure 4:**
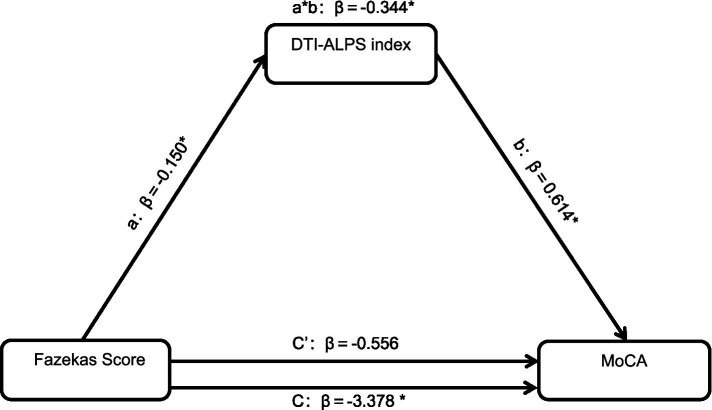
Mediation analysis diagram. Mediation analysis indicated that the association between Fazekas score and MoCA was significantly and partially accounted for by the DTI-ALPS index (effect = −0.344, *p* < 0.05). Abbreviations: MoCA, Montreal Cognitive Assessment; DTI-ALPS, diffusion tensor imaging–along the perivascular space.

Mediation analysis indicated that the association between Fazekas score and MoCA was significantly and partially accounted for by the DTI-ALPS index (effect = −0.344, *p* < 0.05), (Figure 4).

## 4 Discussion

Previous studies have established that T2DM and metabolic syndrome are closely linked to heightened neuroinflammation, oxidative stress, and vascular dysfunction (Oguntibeju, 2019; Bhatti et al., 2022; Domingueti et al., 2016), all of which can impair brain glymphatic system performance (Mogensen et al., 2021; Gędek et al., 2023; Chen et al., 2021). Notably, research has shown that the DTI-ALPS index is reduced in patients with PD and correlates significantly with motor and cognitive dysfunction, suggesting that the degree of DTI-ALPS index reduction reflects disease severity (Bae et al., 2023). Importantly, Alzheimer’s disease and T2DM share overlapping pathological mechanisms, including chronic oxidative stress, insulin signaling dysregulation, neuroinflammation, Aβ accumulation, and tau hyperphosphorylation, which collectively drive disease progression (Chen et al., 2019). These findings provide a strong rationale for investigating the utility of DTI-ALPS in patients with T2DM.

T2DM and sleep disorders exhibit a bidirectional relationship, with glymphatic function being most efficient during sleep (Shen et al., 2022). This interaction creates a vicious cycle, as poor sleep quality in patients with T2DM may further compromise brain glymphatic activity. Jiang et al. (2017) demonstrated a threefold reduction in CSF contrast agent (Gd-DTPA) clearance in T2DM rats compared to controls, indicating impaired glymphatic function, using gadolinium-DTPA-enhanced MRI. Subsequent studies employing DTI-ALPS technology, including those by Yang et al. (2020), Tuerxun et al. (2024), Yu et al. (2024), and Roy et al. (2025), consistently reported significantly lower DTI-ALPS index in patients with T2DM relative to healthy individuals. Our results align with these findings, supporting the hypothesis that glymphatic activity is diminished in T2DM.

We also observed hemispheric asymmetry, with lower DTI-ALPS indices in the left hemisphere compared to the right. This lateralization is consistent with prior research showing that left-hemisphere perivascular spaces (H-EPVS) in patients with T2DM negatively correlate with cognitive performance (Pan et al., 2025), suggesting greater vulnerability of the left hemisphere to diabetic damage. We propose that glymphatic dysfunction may be more pronounced in the left hemisphere of patients with T2DM, though further studies are needed to confirm this hypothesis. Yang et al. (2020) reported significantly lower DTI-ALPS index in patients with a T2DM duration of ≥10 years compared to those with shorter disease duration (<10 years) and HC. Our findings corroborate these observations, reinforcing the potential of the DTI-ALPS index as a biomarker for glymphatic dysfunction and disease severity in T2DM. Additionally, we found that long-standing T2DM was associated with persistently low DTI-ALPS index and MoCA scores, implying that chronic diabetes may lead to widespread brain glymphatic impairment and cognitive decline.

T2DM is typically characterized by insulin resistance and/or insufficient insulin secretion. Insulin resistance, defined as reduced sensitivity of target tissues to insulin, plays an important role in diabetes-related cognitive impairment (Barone et al., 2021). This study investigated the impact of insulin resistance and glymphatic dysfunction on cognitive function in patients with T2DM.

- Firstly, the DTI-ALPS index in patients with insulin resistance was significantly lower than that in patients without insulin resistance.
- Secondly, MoCA scores positively correlated with DTI-ALPS index, while HOMA-IR showed a negative correlation.
- Finally, Mediation analysis indicated that the association between Fazekas score and MoCA was significantly and partially accounted for by the DTI-ALPS index in patients with T2DM.

These findings highlight a close association among insulin resistance, glymphatic dysfunction, and cognitive impairment, collectively constituting a core pathophysiological link. Insulin resistance may act as an upstream driving factor, influencing cognitive status by impairing the glymphatic clearance of metabolic waste. The glymphatic system serves as a key mediator in this process, with its functional status directly related to the occurrence and severity of cognitive decline. Specifically, elevated HOMA-IR is associated with reduced glymphatic activity (reflected by a lower DTI-ALPS index), which in turn correlates with an increased risk of cognitive impairment (indicated by lower MoCA scores). The specific mechanisms of action and supporting clinical evidence are described below.

(i) Insulin resistance directly impairs glymphatic function. The glymphatic system facilitates CSF–ISF exchange via perivascular spaces (PVS), clearing metabolic waste such as Aβ and tau protein (Iliff et al., 2012).
(ii) Insulin resistance disrupts cerebrovascular and metabolic clearance processes through multiple mechanisms. First, it accelerates atherosclerosis, weakening arterial pulsation in the brain. Because arterial pulsation drives CSF flow in PVS (Jessen et al., 2015; Li et al., 2021), reduced pulsation diminishes CSF–ISF exchange efficiency, impairing waste clearance.

Consistent with prior findings by Tuerxun et al. (2024), our study revealed a significant inverse correlation between DTI-ALPS index and HOMA-IR. Specifically, the insulin-resistant group (HOMA-IR ≥ 2.69) exhibited a markedly lower DTI-ALPS index (1.386 ± 0.196) compared to the non-resistant group (1.595 ± 0.301; *p* = 0.015), with a negative correlation between HOMA-IR and the DTI-ALPS index (*r* = −0.326, *p* = 0.045).

These results suggest that progressive insulin resistance correlates with declining glymphatic activity. Although HOMA-IR primarily reflects peripheral tissue insulin sensitivity, emerging evidence indicates that peripheral insulin resistance indirectly affects the central nervous system (Rhea et al., 2022). For instance, insulin resistance downregulates cerebral insulin-degrading enzyme (IDE), a pivotal enzyme for amyloid-beta (Aβ) clearance. Reduced IDE levels promote Aβ accumulation, particularly in PVS. Concurrently, elevated peripheral insulin competes with Aβ for IDE binding, further suppressing Aβ degradation (Wei et al., 2021). The accumulated Aβ then obstructs the perivascular space, directly impeding cerebrospinal fluid flow and contributing to a cycle where insulin resistance exacerbates Aβ buildup, which in turn deteriorates glymphatic function. Additionally, insulin resistance drives microglia, the brain’s resident immune cells, to shift toward a “pro-inflammatory phenotype,” releasing large amounts of inflammatory mediators (e.g., IL-6, TNF-*α*) and impairing their phagocytic function (Van Dyken and Lacoste, 2018; Newcombe et al., 2018). Inflammatory responses disrupt perivascular space integrity, interfering with fluid exchange, while reduced microglial clearance capacity exacerbates metabolic waste accumulation. This study revealed a negative correlation between Fazekas scores (reflecting cerebral small vessel damage and inflammation severity) and the DTI-ALPS index in patients with T2DM (Fazekas 0–1 subgroup: mean DTI-ALPS index 1.559 ± 0.309; Fazekas 2–3 subgroup: 1.372 ± 0.138, *p* = 0.035), confirming the detrimental impact of inflammation on glymphatic function. Furthermore, Mediation analysis indicated that the association between Fazekas score and MoCA was significantly and partially accounted for by the DTI-ALPS index in patients with T2DM, consistent with findings reported by Xu et al. (2024).

Hypothesis (II): This section explores the central role of impaired brain glymphatic system function as a potential driver of cognitive decline. This system plays a pivotal role in maintaining intracranial microenvironmental homeostasis, and its dysfunction contributes to cognitive impairment via two key mechanisms: waste accumulation and neuronal damage. Metabolic waste buildup diminishes the clearance efficiency of the glymphatic system, leading to abnormal deposition of Aβ and tau proteins, a process that establishes a vicious positive feedback loop. Specifically, Aβ disrupts synaptic structure and impairs neuronal signaling, while hyperphosphorylated tau forms neurofibrillary tangles, directly inducing neuronal death (Roy et al., 2025; Busche and Hyman, 2020). Consistent with prior findings by Yu et al. (2024), Roy et al. (2025), and Hu et al. (2025), we observed a strong positive correlation between the DTI-ALPS index and MoCA scores in patients with T2DM (*r* = 0.860, *p* < 0.001). Notably, patients with T2DM and cognitive impairment exhibited significantly lower DTI-ALPS index than those with normal cognition, reinforcing that reduced glymphatic function correlates with more severe cognitive deficits. Beyond waste clearance, the glymphatic system also facilitates transport of glucose and neurotrophic factors such as brain-derived neurotrophic factor (BDNF). Dysfunction compromises nutrient delivery to cognition-critical regions (e.g., prefrontal cortex, hippocampus), resulting in neuronal energy deficits, reduced synaptogenesis, and impaired learning and memory (Cukierman-Yaffe et al., 2021). Animal studies by (Jiang et al., 2017) demonstrated that glymphatic dysfunction in T2DM rats, manifested by a threefold reduction in CSF contrast agent clearance, was directly associated with increased hippocampal neuronal apoptosis, providing further mechanistic evidence. Our study further clarified this relationship through logistic regression analysis, revealing a significant correlation between HOMA-IR and MoCA scores (*p* = 0.025). Importantly, this association was mediated by glymphatic dysfunction: insulin resistance does not directly cause cognitive impairment but contributes to decline by compromising glymphatic activity.

The present study has some limitations. First, the relatively small sample size (especially T2DM-nCI) and single-institution recruitment may limit generalizability. Future studies should expand cohort size and incorporate multicenter data to enhance external validity. Second, our DTI-ALPS assessment employed a single *b*-value approach, which may not fully capture glymphatic dynamics. Multi-*b*-value acquisition strategies could provide more robust and nuanced measurements in future investigations. Finally, cognitive function was assessed solely using the MoCA, which offers global screening but lacks domain-specific profiling. Comprehensive neuropsychological testing—including memory, executive function, attention, and other domains—would yield a more precise characterization of cognitive impairment.

In conclusion, our study findings indicate that glymphatic system dysfunction is closely associated with T2DM and may reflect disease severity. As glymphatic function declines, cognitive impairment progressively worsens. The DTI-ALPS index demonstrates its potential as an imaging marker associated with cognitive performance in type 2 diabetes mellitus (T2DM). These findings may provide reference for clinicians in developing more effective prevention and treatment strategies, thereby helping to maintain brain health and reduce the risk of dementia and Alzheimer’s disease in T2DM patients.

## Data Availability

The datasets presented in this study can be found in online repositories. The names of the repository/repositories and accession number(s) can be found in the article/supplementary material.
